# Non-destructive detection of single-seed viability in maize using hyperspectral imaging technology and multi-scale 3D convolutional neural network

**DOI:** 10.3389/fpls.2023.1248598

**Published:** 2023-08-29

**Authors:** Yaoyao Fan, Ting An, Qingyan Wang, Guang Yang, Wenqian Huang, Zheli Wang, Chunjiang Zhao, Xi Tian

**Affiliations:** ^1^College of Information and Electrical Engineering, Shenyang Agricultural University, Shenyang, China; ^2^Intelligent Equipment Research Center, Beijing Academy of Agriculture and Forestry Sciences, Beijing, China

**Keywords:** viability detection, maize seeds, hyperspectral imaging, YOLOv7 model, 3D convolution neural network

## Abstract

The viability of Zea mays seed plays a critical role in determining the yield of corn. Therefore, developing a fast and non-destructive method is essential for rapid and large-scale seed viability detection and is of great significance for agriculture, breeding, and germplasm preservation. In this study, hyperspectral imaging (HSI) technology was used to obtain images and spectral information of maize seeds with different aging stages. To reduce data input and improve model detection speed while obtaining more stable prediction results, successive projections algorithm (SPA) was used to extract key wavelengths that characterize seed viability, then key wavelength images of maize seed were divided into small blocks with 5 pixels ×5 pixels and fed into a multi-scale 3D convolutional neural network (3DCNN) for further optimizing the discrimination possibility of single-seed viability. The final discriminant result of single-seed viability was determined by comprehensively evaluating the result of all small blocks belonging to the same seed with the voting algorithm. The results showed that the multi-scale 3DCNN model achieved an accuracy of 90.67% for the discrimination of single-seed viability on the test set. Furthermore, an effort to reduce labor and avoid the misclassification caused by human subjective factors, a YOLOv7 model and a Mask R-CNN model were constructed respectively for germination judgment and bud length detection in this study, the result showed that mean average precision (mAP) of YOLOv7 model could reach 99.7%, and the determination coefficient of Mask R-CNN model was 0.98. Overall, this study provided a feasible solution for detecting maize seed viability using HSI technology and multi-scale 3DCNN, which was crucial for large-scale screening of viable seeds. This study provided theoretical support for improving planting quality and crop yield.

## 1 Introduction

Single-seed sowing is a crucial strategy to boost corn production, save seeds, and reduce labor, but it demands high-quality seeds (Li et al., 2017). On October 11th, 2020, a new standard has been released by China, which raises the germination rate index for single-seed sowing from 85% to 93%. The viability is a critical indicator for evaluating the quality and practicality of seed. Assessment of seed viability could ensure each seed has the potential for germination and healthy growth and promotes the popularization of single-seed sowing. This not only facilitates mechanized sowing and reduces the laboriousness of manual interplanting and seedling transplantation, but also significantly reduces the amount of seed used and conserves a considerable amount of seed production area (Liang et al., 2020). Therefore, the determination of seed viability is of utmost importance in reducing the cost and time loss resulting from planting failures and conserving human resources.

Seed viability is a quality characteristic at the individual level rather than a quantitative trait at the population level. Loss of viability among individuals in the same population is not synchronous, making it challenging to detect the viability of single-seed. According to the International Seed Testing Association (ISTA) rules (Association, I.S.T, 1999), common methods for seed viability detection include germination and staining (Cheng et al., 2023). The conventional germination method is the most accurate, but it is time-consuming and requires a lot of material resources. On the other hand, staining is only suitable for a small number of samples. Therefore, it is necessary to develop a rapid-nondestructive technique for single-seed viability detection in large quantities.

In the field of seed quality detection, hyperspectral imaging technology has been widely utilized. However, research on seed viability detection is relatively limited. Jannat Yasmin et al. (2022) presented an online detection system of watermelon seed viability based on longwave near-infrared (LWNIR) HSI, demonstrating its potential application in predicting seed viability. Wang et al. (2021) developed the discrimination models of seed viability using the feature wavelengths and full wavelengths of the visible and shortwave near-infrared (Vis-SWNIR), the result revealed that both models attained an accuracy rate surpassing 95%, suggesting that the seeds with different aging stages exhibited unique spectral features, and the characteristic wavelengths can effectively provide the key information of Zea mays seed quality. Pang et al. (2021) conducted a germination experiment on maize seeds with different aging stages, a 2D convolutional neural network (2DCNN) model was developed by combing deep learning algorithms with hyperspectral technology. The accuracy of this model reached 99.96%, which was significantly higher than machine learning and one-dimensional convolutional neural network (CNN). It was worth pointing out that the model demonstrated a relatively fast convergence speed, which highlighted the feasibility and effectiveness of combining deep learning with hyperspectral technology to determine the viability of single-seed. Ambrose et al. (2016) investigated the feasibility of using HSI technology to differentiate the viability of maize seeds. One group of maize samples was subjected to microwave heat treatment, while the other group served as the control. PLS-DA was employed to classify the heat-treated (aged) and untreated (normal) maize seeds. The results showed that the classification model achieved the highest classification accuracy in the LWNIR region, with calibration set accuracy of 97.6% and prediction set accuracy of 95.6%. These studies achieve high accuracy by predicting the aging level or treatment condition of seeds instead of the actual results of germination experiments. And they mainly rely on overall image information for seed viability classification. However, they overlook the significance of local information within seeds and fail to consider subtle variations and characteristics in different seed regions.

Generally, the evaluation of germination rate of seeds mainly depends on manual labor, which is time-consuming and cumbersome. Zhao et al. (2022) proposed a detection method for the germination rate of rice seeds using deep learning models, which took an average of 0.011 seconds for each image while achieving a mAP of 0.9539, meeting the demands of real-time detection, indicating that the YOLO-r model had great potential for rapidly and precisely determining the germination status of seeds. Bai et al. (2023) developed an improved discriminative approach for the detection of seed germination using YOLOv5. This technique enables the swift evaluation of parameters such as wheat seed germination rate, germination potential, germination index, and average germination days.

The emergence ability of seedlings is crucial for seed growth and crop yield improvement (Cui et al., 2020). In recent studies, significant progress has been made in correlating seed germination ability and seedling growth through various measurement methods. However, traditional manual measurement techniques for assessing parameters like bud length have been found to be inefficient and prone to errors due to the complex and twisted nature of buds. To address this issue, Adegbuyi and Burris (1988) found there was a significant correlation between seed germination ability and seedling growth by measuring comprehensive growth parameters. However, manual measurement method of bud length is inefficient and error-prone due to their curved and twisted nature. Gaikwad et al. (2019) developed a semi-automated tool for measuring leaf length, width, and area. Abdelaziz Triki et al. (2021) used the Mask R-CNN algorithm to effectively segment and measure leaf characteristics and obtained an error rate of around 5%. An enhanced algorithm based on the mask RCNN was introduced by Shen et al. (2023) to recognize defective wheat kernels. The experimental outcomes showed that this refined algorithm facilitated quicker and more precise detection of unsound kernels, effectively tackling issues linked to kernel adhesion. Masood et al. (2021) propose an automated method that utilizes the Mask RCNN model to achieve precise localization and segmentation of brain tumors. Cui et al. (2022) constructed a recognition model using hyperspectral data and feature extraction algorithms to predict maize root length, showing a significant correlation between root length and viability. Therefore, it is of great significance to measure and predict the seed viability using computer technology.

The above study highlighted the significance of seed viability determination and emphasized the need of developing rapid and non-destructive technology for single-seed viability detection. HSI has been established as a useful tool for seed quality detection, and the integration of deep learning and hyperspectral technology can establish an effective seed viability detection model. However, previous studies commonly used relatively simple models, and lacking the prediction model of maize seeds viability developed by 3DCNN and hyperspectral images. This study proposed an improved method for identifying the viability of maize seeds based on germination experiments. The aim of the study is to explore the potential of using hyperspectral images and 3DCNN to identify the viability of maize seeds. Specifically, the objectives are to: (1) select characteristic wavelengths that represent seed viability, (2) combine HSI with 3DCNN to establish the optimal classification model for maize seed viability, (3) evaluate the feasibility of using YOLOv7 model instead of the human eye to determine the seed germination status, (4) evaluate the ability of Mask R-CNN in bud segmentation and bud length prediction.

## 2 Materials and methods

### 2.1 Maize sample preparation

#### 2.1.1 Aging experiment

Due to the high quality and the resistance to multiple stressors, “Jingke 968” maize is extensively cultivated in eastern and northern China. Therefore, it was selected as the experiment sample in this study. To ensure the accuracy of the experiment, seeds with uniform size and shape were manually selected, then all seeds were disinfected by soaking them in a 0.5% sodium hypochlorite solution for 5 minutes, followed by rinsing with distilled water five times, and air-dried under natural conditions.

To simulate the natural aging process of seeds, the experiment samples were artificially aged. All seeds were exposed to high temperature and high humidity conditions (45 °C and a relative humidity of 95%) and stirred twice a day to ensure uniform exposure (Zhang et al., 2020). 150 maize seeds were taken out randomly at aging 2, 4, 6, and 8 days, respectively. Additionally, 150 untreated seeds were selected as the control group (CK). Therefore, a total of 750 maize seeds within five aging stages were obtained and used for subsequent experimentation.

#### 2.1.2 Hyperspectral imaging system

**Figure 1:**
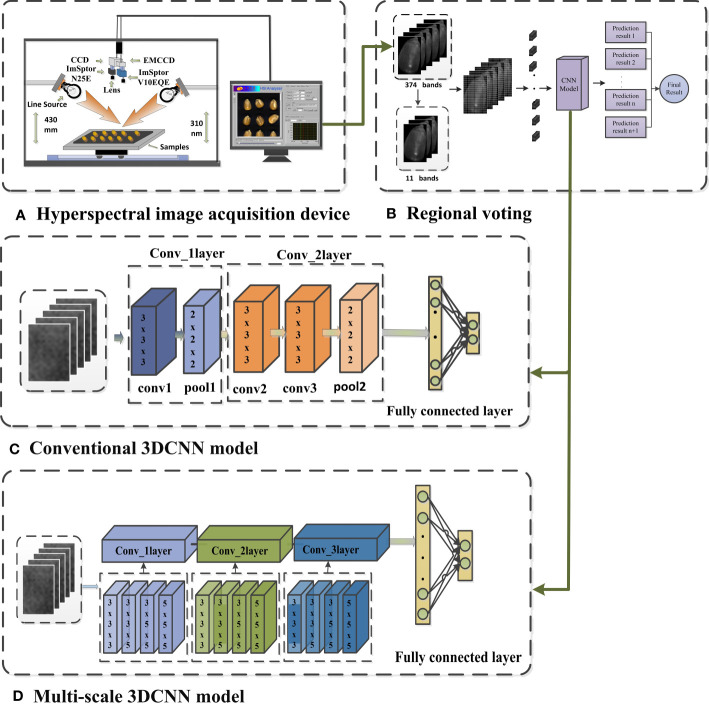
Diagram of the 3DCNN for hyperspectral image classification **(A)** Hyperspectral image acquisition device, **(B)** Regional voting, **(C)** Conventional 3DCNN model, **(D)** Multi-scale 3DCNN model.

Two HSI systems, the Vis-SWNIR and LWNIR, have been built in the Intelligent Detection Laboratory of the China Agricultural Equipment Technology Research Center (Fan et al., 2018). The Vis-SWNIR system is capable of acquiring hyperspectral images within the wavelength range of 327-1098 nm, encompassing 1000 spectral variables, while the LWNIR system can capture images within the range of 930-2548 nm, containing 256 spectral variables. The Vis-SWNIR system includes an imaging spectrometer, an electron-multiplying charge-coupled device camera with a resolution of 502×500, a camera lens, and a spectraCube data acquisition software. Similarly, the LWNIR system includes an imaging spectrometer, a charge-coupled device camera with a resolution of 320×256, a camera lens, and a spectral acquisition software (Tian et al., 2021). And the acquisition software of both systems was developed using LabVIEW (National Instruments Inc., Austin, TX, USA) to facilitate the acquisition of spectral images, as well as to manage the camera and motor operations. Both systems share two 300-watt halogen lamps to provide stable illumination. In addition, an electrically operated moving platform and a computer are available for sample placement (Capable of accommodating up to 96 samples simultaneously) and hyperspectral image acquisition (**Figure 1A**) (Liu et al., 2022).

To ensure the accuracy and reliability of the hyperspectral images (*E_raw_*), calibration operation is essential to eliminate the effects of uneven illumination of the light source and camera dark current changes (An et al., 2022). The calibration operation involved using a white reflection board (with a reflectance of 99%) (*E_w_*) to acquire a standard white reference image in the same sampling environment as the sample, while turning off the light source and covering the lens to obtain a black reference image (with a reflectance of 0%) (*E_d_*). The calibrated image can be calculated using the following formula:


(1) $$E_{c}=\frac{E_{raw}-E_{d}}{Ew-Ed}$$

After calibration, in the Vis-SWNIR region, a subset of 347 spectral variables within the 420-1000 nm range was selected for further analysis, considering the abundance of spectral data and the presence of duplicate information in adjacent spectra. On the other hand, in the near-infrared region, due to the limited number of available bands, all spectral variables (256) were directly included in the analysis. To separate maize seeds from the background, a mask was applied to segment the hyperspectral image. The gray-scale images at 801 nm and 1098 nm were selected as the mask images for the Vis-SWNIR and LWNIR bands, respectively. The average spectral curves were obtained by calculating the mean reflectance under the mask. Lastly, in order to eliminate the influence of the instrument, the Savitzky-Golay (SG) and Standard Normal Variate (SNV) methods were utilized to preprocess the spectra.

#### 2.1.3 Standard germination test

**Figure 2:**
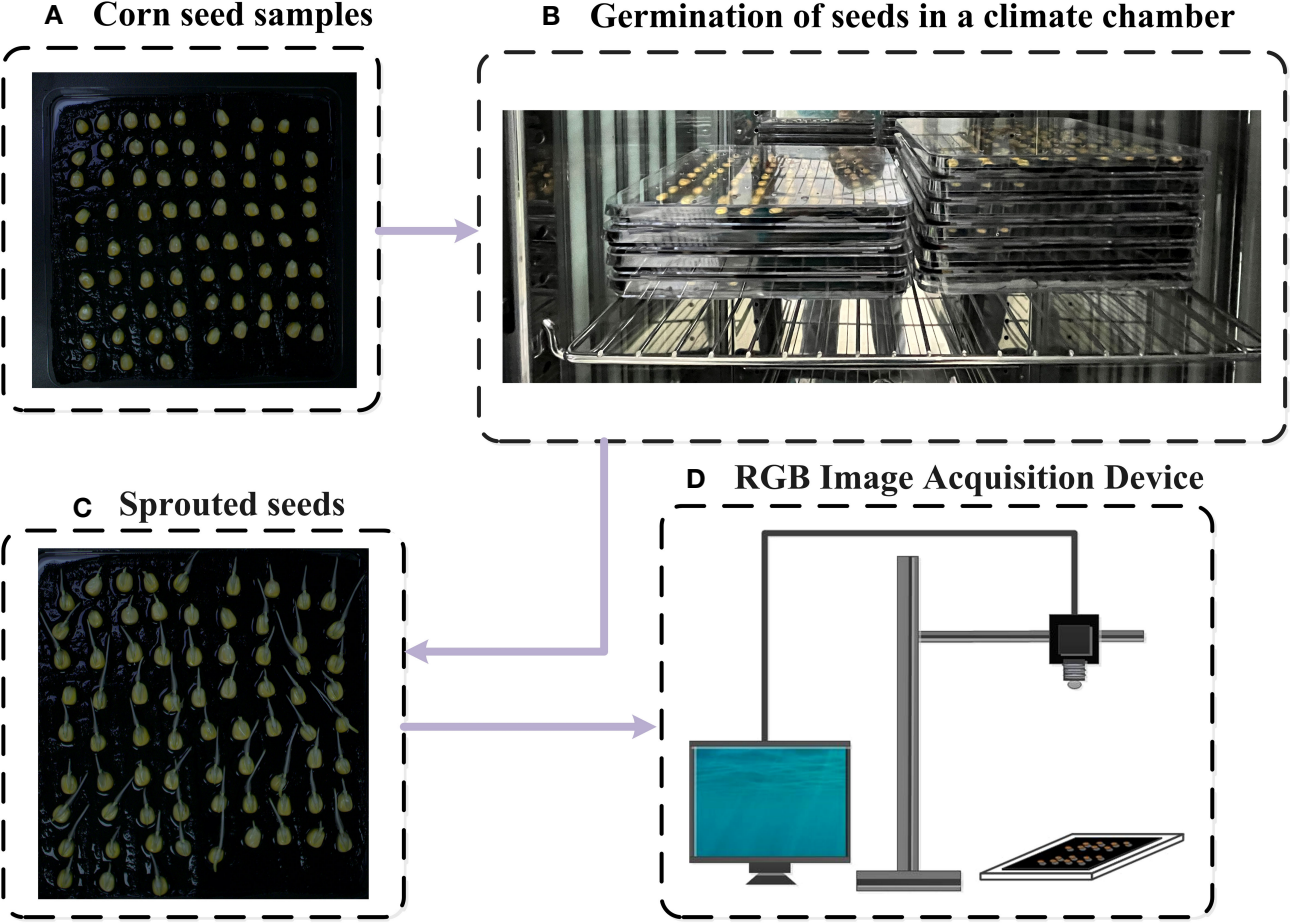
Diagram of the standard germination experiment **(A)** Corn seed samples, **(B)** Germination of seeds in a climate chamber, **(C)** Sprouted seeds, **(D)** RGB iamge acquisition device.

A transparent box measuring 25cm×25cm was used as a germination chamber, and 75 seeds were placed in each box. A total of 10 boxes were used in the experiment. Prior to the germination test, the germination boxes were sterilized with 75% ethanol (Suksungworn et al., 2021), and three layers of gauze were placed in each germination box to provide continuous moisture for the seeds. A black gauze was placed on the top layers as the background for photography (**Figure 2A**). An equal amount of distilled water was added to each box, and the temperature was set to 25°C with 12-hour intervals of light and dark (**Figure 2B**). Throughout the 7-day germination experiment (Long et al., 2022), the germination progress of maize seeds was monitored daily at specific time intervals. According to the ISTA standard, the germination rate was determined (Wang et al., 2022c).

#### 2.1.4 RGB image acquisition

RGB images of maize seeds were captured using BASLER industrial cameras (acA1920-25um/uc, BASLER AG, Germany, 2.4 MP,100 fps) during germination test (**Figure 2D**) (Shen et al., 2023). An adjustable camera platform was built to ensure consistency of the images and prevent camera shake. The position of the germination box relative to the lens was kept fixed during each image capture. Indoor lighting was turned on and curtains were drawn for each capture. After placing the seeds into the boxes (Day 0), images of each box were immediately captured. Subsequently, images were captured every 15 hours for 7 consecutive days (**Figure 2C**). The dataset used in this study consisted of a total of 3000 maize seeds (All the captured RGB images collectively contain 3000 seeds). Among them, 2250 seeds were designated as training samples, while the remaining seeds were allocated to the test set.

### 2.2 Data processing

#### 2.2.1 Successive projections algorithm

Hyperspectral data typically consists of numerous bands, and certain bands may exhibit high correlation or contain redundant information (Han et al., 2022).When training 3DCNN with full-band data, it will lead to a significant increase in the number of networks training parameters, resulting in a more complex model. This phenomenon is commonly referred to as the curse of dimensionality. (Köppen, 2000). However, band selection (Sun and Du, 2019) allows retaining spectral bands that are closely related to seed vigor assessment while removing irrelevant bands, thereby enhancing the feature extraction and discriminative capabilities of the model.

Additionally, the use of dimensionality reduction data sets can effectively reduce the complexity of the model, mitigating the risk of overfitting and enhancing the model’s generalization ability and stability (Aloupogianni et al., 2023). Moreover, fewer computing resources are required during model training and inference, leading to a significant improvement in the computational efficiency of the model (XingJia et al., 2022).

Successive projections algorithm is a classical band selection method that can map high-dimensional spectral data to a low-dimensional space through multiple projections(de Almeida et al., 2018). SPA is a forward iterative search method used for selecting spectral information with minimal redundancy to address collinearity issues. The steps of SPA are shown in **Table 1**. The SPA is widely used in hyperspectral image processing attributed to its advantages of fast computation speed and easy implementation (Chen et al., 2023b). Therefore, the SPA was used in this study to perform feature selection on the processed average spectra of Vis-SWNIR and LWNIR, in order to perform dimensionality reduction on the hyperspectral data.

**Table 1:** Successive projections algorithm.

Input:Dataset with features and target variable Output:Feature subset for analysis	
**Step 1:** For each feature in the dataset: a. Compute projection coefficients with respect to the target variable. b. Store the computed coefficients.
**Step 2:** Initialize an empty feature subset. **Step 3:** Repeat until desired subset size is reached or stopping criterion met: a. Find the feature with the maximum projection coefficient. b. Add the selected feature to the feature subset. c. Project out the influence of selected features on remaining features. d. Recalculate projection coefficients of remaining features.

#### 2.2.2 Machine learning

Support Vector Machine (SVM) (Cortes and Vapnik, 1995) is a powerful algorithm for classification and regression that finds an optimal hyperplane to separate data points of different classes. It handles high-dimensional datasets, avoids overfitting, and can handle non-linear problems using kernel functions. K-Nearest Neighbor (KNN) (Zhang, 2022) is a basic algorithm that selects the K nearest samples based on their feature values and uses their labels as predictions. Subspace Discriminant Analysis (SDA) (Zhao and Phillips, 1999) is a pattern classification method that aims to find a low-dimensional subspace to maximize the separation between different classes. In this study, the aforementioned machine learning methods were used to classify the viability of maize seeds at different aging stages for optimal classification accuracy.

#### 2.2.3 Deep convolutional neural network

The CNN combines the concepts of convolutional filtering and neural networks by utilizing local receptive fields and weight sharing to reduce the number of network parameters and speed up model training (Ghaderizadeh et al., 2021). Compared to the widespread use of two-dimensional convolution, three-dimensional convolution is less commonly used in practice. However, HSI contain rich spectral information, and using two-dimensional convolution may make the interband correlations of HSIs underutilized (Ge et al., 2020). To address this issue, this study introduced a 3DCNN, which can thoroughly extract feature relationships across different feature channels (**Figure 1C**), thereby enabling it to concurrently extract integrated spectral and spatial features from hyperspectral imagery (Sun et al., 2022).

Before inputting hyperspectral images into the network, standardization is performed to ensure that the data is within the same scale and range, enabling the network to learn weights faster and converge more easily during training. Moreover, data standardization can help avoid the problems of gradient disappearance or explosion, and improve the stability and generalization ability of the network. To obtain multiple convolutional features of HSI, multi-scale convolution is employed in the same convolutional layer, which can acquire both global and local information. Four different convolution kernels of 3×3×3, 3×3×5, 3×5×5, and 5×5×5 were selected to extract feature information and fused on the channel. This method can enhance the classification accuracy of the model. As illustrated in **Figure 1D**, each convolution kernel in the first convolution module has 16 filters, each kernel in the second convolution module has 32 filters, and each kernel in the third convolution module has 64 filters. The activation function in the three-dimensional convolution module uses Rectified Linear Unit (RELU) and is compressed by the pooling layer to reduce the amount of data and parameters, as well as alleviate the overfitting phenomenon. To ensure that the features extracted by different convolution kernels in the same module can be effectively connected, different parameters need to be set according to different situations, such as stride and padding. Finally, the output is produced through 1 fully connected layer and 1 output layer, and the output layer employs the SoftMax activation function.

To extract features from hyperspectral images of maize seeds at a more microscopic level and increase the amount of data, a window size of 5×5 was selected for segmentation (**Figure 1B**). To eliminate the influence of background on classification, small blocks containing 0-pixel points were discarded. As the size of maize seeds varies, the number of blocks obtained from different segments of maize seeds is also inconsistent. To address this issue, this study employed a majority principle labeling aggregation method, as **Table 2**.

**Table 2:** Majority principle labeling aggregation method.

Input: Segmented maize seed blocks Output: Predicted potential for germination of maize seeds	
Step 1: Initialize: a. Assign Label 1 to represent potential for germination. b. Assign Label 2 to indicate maize grain block affiliation.
Step 2: For each segmented maize seed block: a. Feed the block into the model for prediction of its potential for germination. b. Store the prediction result.
Step 3: For each maize seed: a. Retrieve predictions of multiple small blocks belonging to the same maize seed. b. Count the number of correct predictions. c. If more than half of the predictions are correct: - The predicted result of the maize seed is deemed correct.

In this study, the germination experiment showed that 404 viable samples and 346 nonviable samples were collected from 750 seeds. Given that the hyperspectral images were collected in a sequential manner based on the aging gradients of the seeds, it was crucial to maintain a balanced distribution of germinated and non-germinated samples in the test set. Therefore, a representative test set was carefully selected, consisting of 75 seeds, including the first seed, the 10th seed, the 20th seed, and so on. The remaining 675 seeds were allocated for the training phase. Through this meticulous approach, it was ensured that the test set encompassed samples from diverse categories, enabling an accurate evaluation of the classification model’s performance.

#### 2.2.4 Establishment of Mask R-CNN model for bud length detection

**Figure 3:**
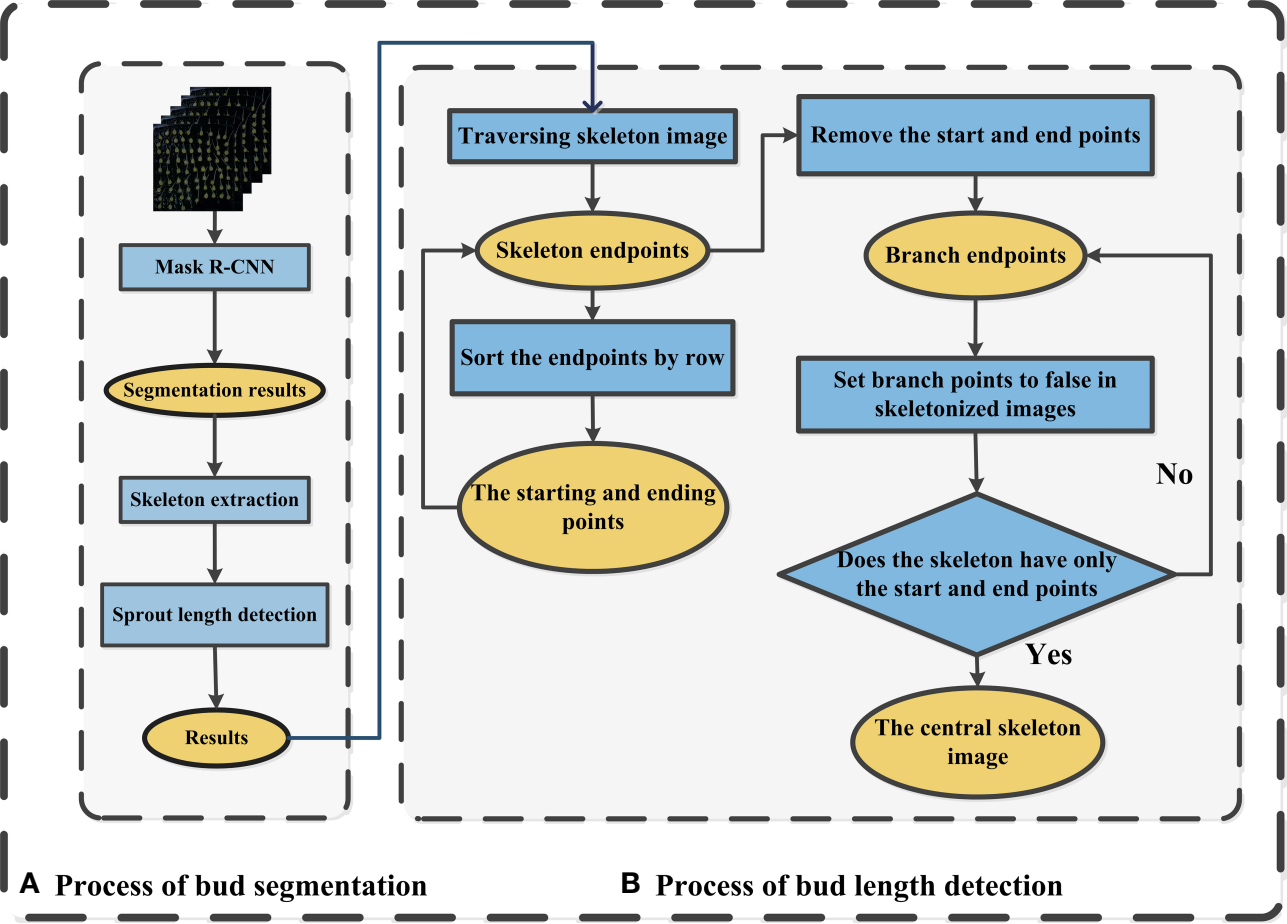
Diagram of the maize bud length detection process **(A)** Process of bud segmentation, **(B)** Process of bud length detection.

In order to measure the length of maize seed bud, the Mask R-CNN (He et al., 2017) (With resnet50_fpn as backbone) model was utilized to segment the bud from single-seed image firstly, then a skeleton extraction algorithm was applied to extract the skeleton of the bud (**Figure 3A**). Next, the bud length detection algorithm was used to remove the branches in the skeleton for obtaining the central skeleton image. Finally, the actual bud length was calculated by converting pixels to actual length (**Figure 3B**).

Mask R-CNN is a deep learning model that combines object detection and instance segmentation. It extends Faster R-CNN by generating binary masks for each region of interest (ROI), achieving pixel-level segmentation. The network consists of three main components: a backbone network, a Region Proposal Network (RPN) responsible for generating candidate object regions, and two parallel branches dedicated to object detection and mask prediction. Mask R-CNN excels in instance segmentation, object detection, and keypoint detection, making significant contributions to computer vision advancements (Casado-García et al., 2019). The model employs a multi-task loss function, comprising classification loss (*L_cls_*), bounding box loss (*L_bbox_*), and predicted mask loss (*L_mask_*), as represented by equations (2) to (5) (Cong et al., 2023).


(2) $${\text{L = L}}_{\text{cls}}+\;{\text{L}}_{\text{Lbox}}\;+\;{\text{L}}_{\text{mask}}$$

(3) $${\text{L}}_{\text{cls}}\;=\;\sum_{\text{i}}-\log\left[{\text{p}}_{\text{i}}{\text{pi}}_{*}+\left(1-{\text{pi}}_{*}\right)\left(1-{\text{p}}_{\text{i}}\right)\right]$$

(4) $${\text{L}}_{\text{bbox}}\frac{1}{{\text{N}}_{\text{reg}}}\sum_{\text{i}}{\text{pi}}_{*}\text{R }{\text{(t}}_{\text{i}}-\;{\text{ti}}_{*})$$

(5) $${\text{L}}_{\text{mask}}\;=\;-\frac{1}{{\text{m}}^{2}}\sum_{1\leq \text{ i},\text{ j}\leq \text{ m}}\left[{\text{y}}_{\text{ij}}^{*}\log{\text{y}}_{\text{ij}}+\left(1-{\text{y}}_{\text{ij}}^{*}\right)\log\left(1-{\text{y}}_{\text{ij}}\right)\right]$$

*L_cls_* measures the deviation between predicted and actual values for overall accuracy assessment. *L_bbox_* quantifies the disparity between predicted and actual position parameters, assessing the model’s accuracy in bud localization. *L_mask_* evaluates the model’s confidence in pixel-level classification using binary cross-entropy. Combining these components into a multi-task loss function allows for comprehensive evaluation across multiple tasks, resulting in enhanced overall performance.

The skeleton extraction algorithm is a technique used to extract the central line or skeleton of an object in a binary image (Fu et al., 2023). By progressively shrinking connected regions within the object contour, the algorithm produces a concise contour that provides valuable information for image processing tasks like recognition and matching. Various algorithms, such as Zhang-Suen, Morphological Thinning, and Medial Axis Transform, can be employed for this purpose. The Medial Axis Transform (MAT) algorithm, specifically, extracts the object’s central line by iteratively dilating boundary pixels and identifying the nearest internal pixels as skeleton pixels. This process continues until the skeleton pixels stabilize, resulting in a stable and versatile representation suitable for subsequent image processing tasks. The MAT algorithm handles different object shapes and can process grayscale information within binary images. Seed germination images exhibit a wide range of shape features, such as bud length, curvature, and angle. However, traditional methods for measuring bud length rely on manual measurements, which are time-consuming and prone to significant subjective biases. The MAT (Medial Axis Transform) skeleton extraction algorithm was chosen to obtain the central line of buds. However, the resulting skeleton may contain branches that need to be eliminated to derive the center skeleton. The process of centerline skeleton extraction is illustrated in the following **Figure 3B**.

In this study, a transparent box with a side length of 250 mm was used as a reference to convert pixels to actual lengths in millimeters. The calculation formula is:


(6) $$\text{Ratio }=\;L_{box}/1164$$

Here, *L_box_* represents the side length of the transparent box, and 1164 is the number of pixels corresponding to the transparent box in the image. According to the calculation formula, it can be derived that one pixel corresponds to 0.215 mm.

#### 2.2.5 Establishment of YOLOv7 model for seed germination detection

The seed quality detection methods such as germination and staining techniques are time-consuming and rely heavily on human intervention, which may lead to inaccurate results due to human error. In order to develop an automated and standardized method for detecting seed germination that is efficient, accurate, and reliable, the YOLOv7 (Wang et al., 2022b) object detection algorithm was selected in this study, which is one of the most widely used algorithms for object detection since its release in 2015 (Dewi et al., 2023). YOLOv7 is a real-time object detection algorithm (Soeb et al., 2023), which has evolved from YOLOv5 and has faster inference speed, improved detection accuracy, and reduced computational complexity. The algorithm consists of three main parts: the input layer, backbone layer, and output layer (Tang et al., 2023), and uses either a loss function with or without an auxiliary training head (Zhou et al., 2023).

The loss function is used to update the gradient loss during the training process (Cai et al., 2023). The YOLOv7 algorithm is evaluated using various metrics such as precision, mAP, recall, and F1 score (Zhao et al., 2023), and curves such as the F1-Confidence curve, precision-confidence curve, recall-confidence curve, and precision-recall curve are used to optimize the algorithm’s performance and achieve the best balance between precision and recall.

This study utilized a self-built dataset of maize seeds, comprising images of seeds from various angles and sizes, each with corresponding labels in YOLO format. The data collection and preprocessing process was conducted using the same method as Mask R-CNN. The dataset used in this study consisted of a total of 7000 maize seeds. Among these, 4200 seeds were designated as training samples, 1400 seeds were allocated for the test sets, and the remaining seeds were assigned to the validation sets. To enhance the accuracy and robustness of the model, the YOLOv7.pt (https://github.com/WongKinYiu/yolov7) pretrained weights provided by the official website were employed for training. These weights were trained on a large-scale dataset, which can significantly reduce the training time while improving the training effect. The Adam optimizer, a widely used optimizer that can optimize at different learning rates, was used to update the model parameters during training. The parameters of the Adam optimizer were adjusted based on the size of the learning rate in the training process to achieve better training results. A batch size of 2 and a training iteration of 300 were used in this study.

## 3 Results and discussion

### 3.1 Seed germination result

**Figure 4:**
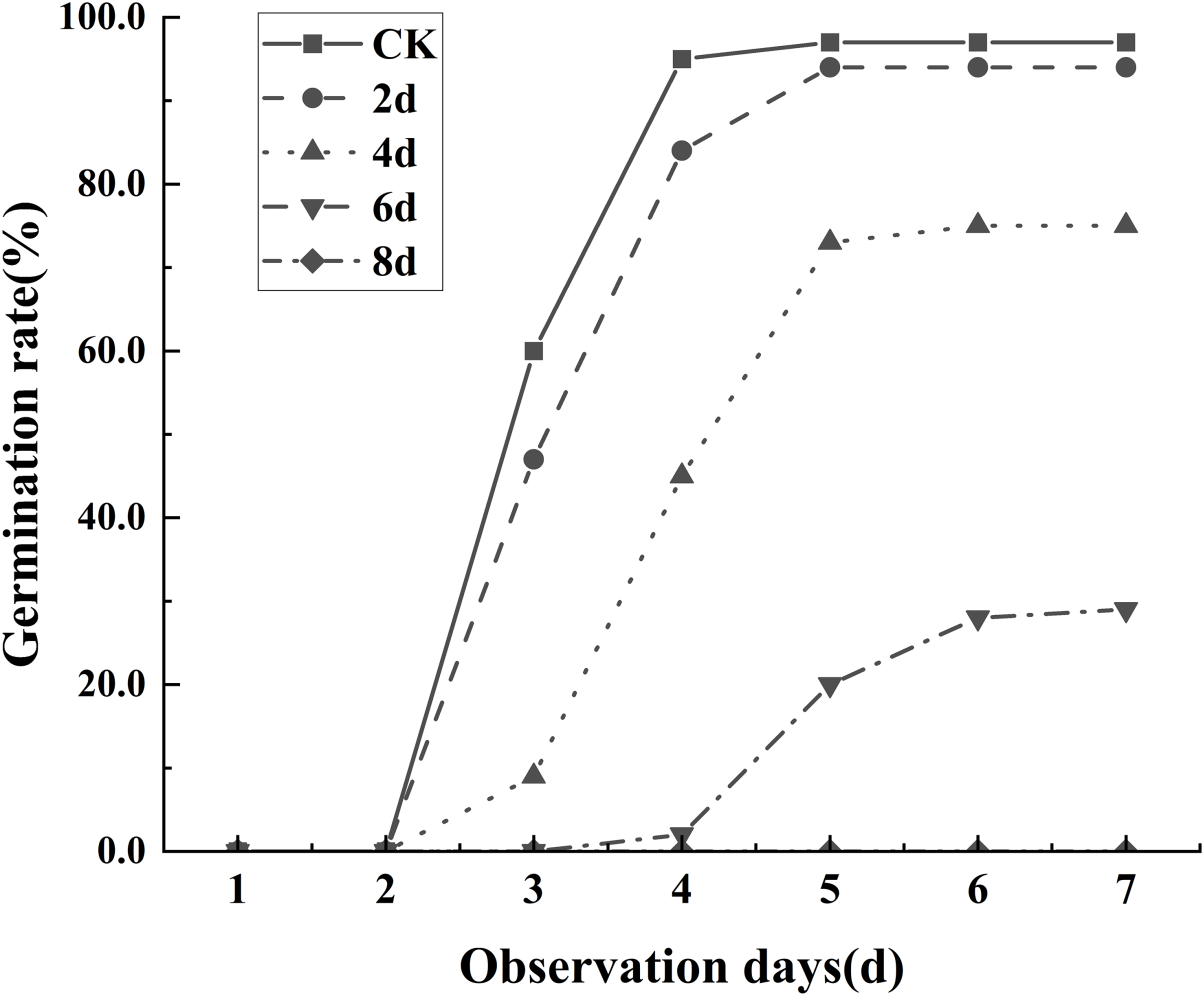
Germination levels of seeds at different aging times.

The experimental results showed that the degree of seed aging was significantly correlated with the germination rate. As shown in the **Figure 4**, on the seventh day of observation, all seeds that were not aged can germinate, and only a few seeds that aged for 2 days failed to do so. Most seeds that aged for 4 days still retained their viability, with only a few seeds that aged for 6 days able to germinate. Seeds that aged for 8 days experience almost complete mortality. Thus, it can be inferred that seed aging leads to a decline in the germination rate, and the more prolonged the aging process, the more apparent the decline in the germination rate.

### 3.2 Average spectrum

**Figure 5:**
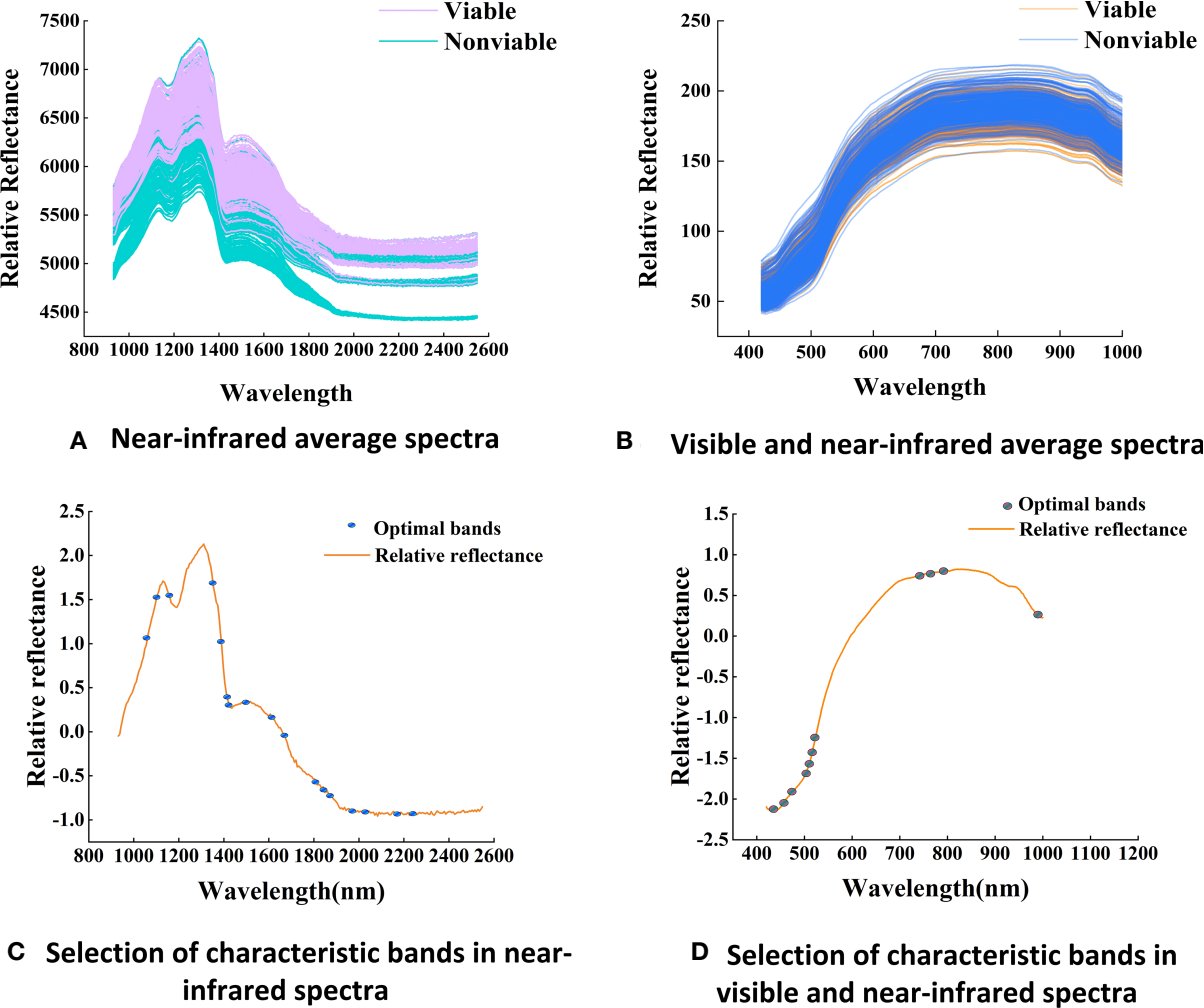
Average spectra and the distribution of optimal bands **(A)** Near-infrared average spectra, **(B)** Visible and near-infrared average spectra. **(C)** Selection of characteristic bands in near-infrared spectra, **(D)** Selection of characteristic bands in visible and near-infrared spectra.

**Figure 6:**
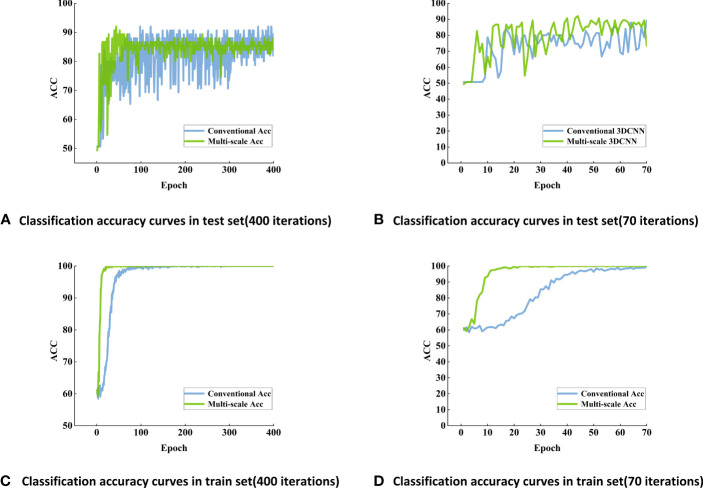
Classification accuracy curves of maize seed viability based on conventional 3DCNN models using Vis-SWNIR hyperspectral image **(A)** Classification accuracy curves in test set (400 iterations), **(B)** Classification accuracy curves in test set (70 iterations), **(C)** Classification accuracy curves in train set (400 iterations), **(D)** Classification accuracy curves in train set (70 iterations).

By analyzing the spectral curve features (**Figures 5A, B**), it is easy to observe that the spectral reflectance of both wavelength regions increased with the decreasing of maize seed viability, indicating that the light absorption capacity of maize tissue increases with the aging degree. The spectral curves are monotonic in the Vis-SWNIR region, with the average spectral curve gradually increasing in the 400-800 nm region and then slowly decreasing. However, in the LWNIR region, the spectral curve is more complex, capturing two distinct reflection peaks located around 1100 nm and 1300 nm, respectively. The former could potentially be associated with the presence of C-H bonds in lipids, while the latter could be described as a combination of the first overtone of N-H stretching along with the fundamental N-H in-plane bending and C-N stretching with N-H in-plane bending vibrations (Wang et al., 2022d).The spectral curve characteristics can be used to discriminate maize seeds with different germination potentials. As shown in **Figure 6**, the spectral data of maize seeds with different viability have similar trends in the Vis-SWNIR and LWNIR regions. However, in the Vis-SWNIR region, these curves are basically mixed together, making it difficult to distinguish clearly. In contrast, there are significant differences in the LWNIR region, which may be related to the breakdown of chemicals during the aging process of organisms. Nevertheless, some mixed situations still exist, indicating that it is difficult to distinguish the seeds with or without viability according to the average spectra of hyperspectral image.

### 3.3 Key wavelength selection of maize seed viability

During the aging process of maize seeds, a series of changes occurs in the internal chemical substances (Xin et al., 2011), with the extent of these changes depending on the degree of seed vitality. These chemical substances include stored energy and nutrients, such as starch, proteins, and lipids (Xu et al., 2022). Proteins may undergo degradation, leading to the release of amino acids and structural damage to proteins. At the same time, the lipid content in the seed gradually oxidizes, resulting in lipid decomposition and the generation of free radicals, thereby affecting the seed’s metabolism and viability. Additionally, starch gradually degrades into soluble sugars. This difference is the main reason for spectral changes during the aging process. After SG and SNV preprocessing, 18 and 11 characteristic bands were extracted from the Vis-SWNIR region and LWNIR region (**Figures 5C, D**). These characteristic bands were located at the peaks and valleys of the spectrum, reflecting the changes in water content and protein levels of the seeds.

### 3.4 Maize seed viability detection based on full-wavelength spectra and machine learning

By analyzing the classification accuracy obtained from SVM and Ensemble analysis, there was no significant difference between Vis-SWNIR and LWNIR regions in predicting seed viability (**Table 3**). However, KNN exhibited slightly higher accuracy with LWNIR, indicating its greater universality and better performance in detecting seed viability. However, due to the minimal differences between seeds with adjacent aging gradients (Feng et al., 2018), particularly those seeds that aged for 4 days and 6 days, these distinctions may not be immediately discernible, presenting a challenge in accurately determining the germination potential of seeds with similar levels of aging. The germination experiment also showed that the seeds with relatively mild aging did not have inherent germination trends and were easily misclassified by the prediction model. This discrepancy may arise from the fact that maize seeds may not exhibit overt phenotypic changes across different stages of aging (Wang et al., 2022e). However, in actuality, mRNA molecules associated with protein synthesis undergo oxidation through physiological mechanisms. More specifically, research unveiled significantly elevated expression levels of mature enzyme genes and ribosomal protein genes in embryonic roots and shoots as compared to other parts(Wang et al., 2022a). This obstruction hampers protein synthesis, consequently impeding the normal physiological functions of the seeds.

**Table 3:** The classification result of maize seed viability based on full-wavelength spectra and machine learning.

Models	Vis-SWNIR		LWNIR	
	Train set	Prediction set	Train set	Prediction set
SVM	89.3%	83.9%	84.0%	83.3%
KNN	72.0%	69%	85.3%	77.8%
Ensemble	92%	82.4%	85.3%	82.5%

### 3.5 Maize seed viability detection based on key wavelength and 3DCNN model

After 70 training epochs on the Vis-SWNIR hyperspectral images, the accuracy of the training set has stabilized at a high level of 100% (**Figure 6B**), and the accuracy of the test set has also reached its peak. In order to further validate the stability and robustness of the model, the number of training epochs was increased to 400. After 400 iterations, the accuracy of the training set remained at around 100%, while the accuracy of the test set remained at around 90% (**Figures 6A, C**).

By using 3DCNN to process the data, not only the spectral information was considered (Wu et al., 2021), but also the image information was integrated, making the evaluation of maize seed quality more comprehensive and accurate (Collins et al., 2021). Compared with machine learning methods that using all spectral bands as input data, the 3DCNN method only used few representative bands. Traditional machine learning methods tend to lose a lot of information, while the 3DCNN method used in this study can learn more complex features and achieved higher accuracy with fewer bands, with an average accuracy increase of 7 percentage points (**Table 4**). It was worth noting that 3DCNN performs better on the test set and converges faster, which indicated that 3DCNN was an effective method for seed viability detection and had advantages over machine learning classification method in dealing with such problems.

**Table 4:** The classification performance of the maize seed viability based on 3DCNN.

Block		Models			
		Multi-3DCNN		Conventional-3DCNN	
		Vis-SWNIR	SWNIR	Vis-SWNIR	SWNIR
	5 pixels×5 pixels	90.67%	90.67%	92.00%	88%
Split	10 pixels×10 pixels	92.00%	87.33%	92.00%	85.33%
	20 pixels×20 pixels	85.33%	79.00%	86.67%	78.67%
No-split		80.80%	78.50%	79.60%	77.50%

Conventional 3DCNN and multi-scale 3DCNN exhibit different characteristics. Traditional 3DCNN can achieve high accuracy, but they often exhibit slower convergence compared with multi-scale 3DCNN (**Figure 6D**). Multi-scale 3DCNN incorporated convolutional layers with different-sized kernels and pooling layers, allowing the network to process features of varying scales simultaneously (Lin et al., 2020). This enhanced the network’s robustness and improved its tolerance to noise, distortion, and artifacts in the data, and ultimately led to a faster convergence. In addition, the stability of conventional 3DCNN may not be satisfactory and may exhibit some fluctuations and instability. In contrast, multi-scale 3DCNN perform better, possibly due to their utilization of multi-scale convolutional kernels, enabling them to extract more abundant feature information (Shi et al., 2021) (**Figure 6A**). Furthermore, the block-based method effectively increased the amount of data and helped to alleviate overfitting. In the final discrimination, this study adopted a majority principle labeling aggregation method to improve the discrimination accuracy (**Table 4**). To explore the optimal block size, several experiments were conducted, the input images were segmented into different block sizes, including 5 pixels ×5 pixels, 10 pixels ×10 pixels, and 20 pixels × 20 pixels. As shown in **Table 4**, the model achieved a relatively high overall accuracy when 5 pixels ×5 pixels was used. This suggested that the small blocks with 5 pixels ×5 pixels size can effectively capture more local features of the seedy and provides more discriminative information. Conversely, larger blocks may result in information blurring and confusion, thereby impacting the classification accuracy. Consequently, the block-based method with 5 pixels ×5 pixels was finally selected to enhances the detection accuracy of seed viability.

### 3.6 Maize seed germination detection based on YOLOv7 model

**Figure 7:**
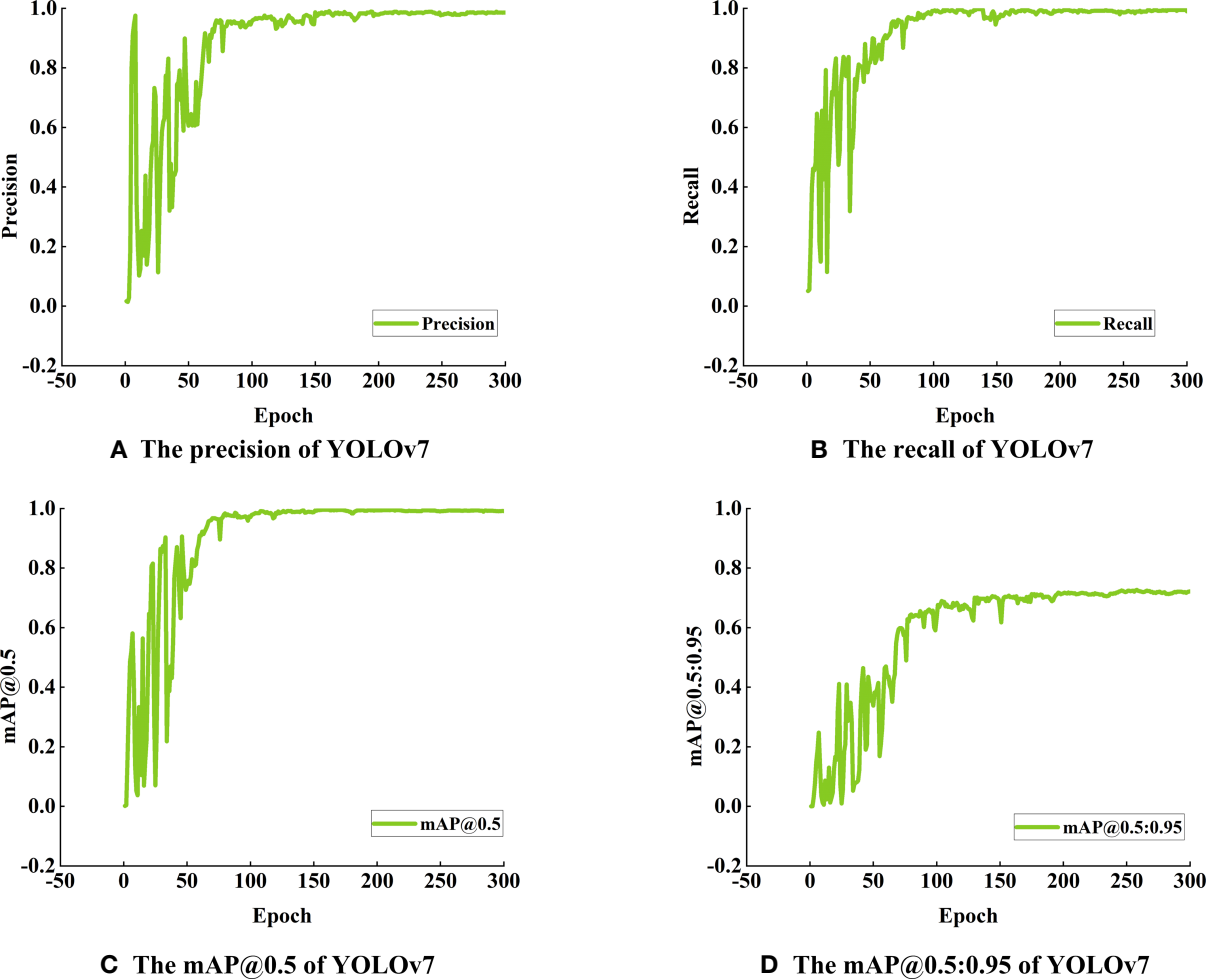
Detection performance of YOLOv7 model for maize seed germination **(A)** The precision of YOLOv7, **(B)** The recall of YOLOv7, **(C)** The mAP@0.5 of YOLOv7, **(D)** The mAP@0.5:0.95 of YOLOv7.

**Figure 7** shows the detection results of germinated maize seeds using the YOLOv7 model, demonstrating its remarkable precision and recall rates of 99.7% and 99.0%, respectively. Additionally, the model achieves a mAP of 99% when applying an Intersection over Union (IoU) threshold of 0.5. Furthermore, the mAP, calculated across a range of IoU thresholds from 0.5 to 0.95, reaches a value of 71%.

In **Table 5**, the YOLOv7 model exhibits an impressive F1 score (The F1 score balances precision and recall, providing a comprehensive evaluation of model accuracy) of 0.99 on all target categories with a confidence threshold set at 0.663, highlighting its exceptional detection performance. Consequently, the YOLOv7 model can achieve both high precisions, accurately identifying true positive predictions, and high recall, effectively capturing all relevant targets during detection. With a confidence threshold set to 0.896, the YOLOv7 model achieves a perfect precision accuracy of 100% for the target categories. This noteworthy precision metric showcases the model’s ability to correctly identify true positive predictions among all the positive predictions made, indicating its reliability and precision in detecting target objects. The model impressively achieves a recall rate (The recall rate quantifies the model’s ability to correctly identify positive targets) of 1.00 with a confidence threshold set to 0.000, indicating that it accurately detects all targets of all categories without any missed detections. This ideal performance underscores the model’s high accuracy and proficiency in target detection tasks Additionally, the model exhibits an mAP (The mAP commonly used to evaluate object detection algorithms’ accuracy and robustness) of 0.991 for all target categories when applying an Intersection over Union (IoU) threshold of 0.5. This further demonstrates the model’s superior detection capabilities across various categories, affirming its exemplary performance.

**Table 5:** The detection result of YOLOv7 model for maize seed germination.

YOLOv7 Training Indicators					
**All classes**	**F1-confidence**	**F1**	0.99	**Confidence**	0.663
	**Precision-confidence**	**Precision**	1.00		0.896
	**Recall-confidence**	**Recall**	1.00		0.000
	**Precision-recall**	0.991 mAP@0.5			


(7) $$R\;=\frac{TP}{TP+FN}$$

(8) $$\text{AP }=\int_{0}^{1}P\left(R\right)dR$$

(9) $$\text{F}1\;=\;\frac{2*P*R}{P+R}$$

(10) $$\text{P }=\;\frac{TP}{TP+FP}$$

(11) $$\text{mAP }=\;\frac{1}{n}\sum_{i=1}^{n}AP$$

In these formulas, True Positives (TP) represent the number of samples where the predicted label is positive and the actual label is also positive. T represents the total number of samples, and False Negatives (FN) indicate the number of samples where the predicted label is negative, but the actual label is positive. Similarly, False Positives (FP) represent the number of samples where the predicted label is positive, but the actual label is negative. Moreover, the area under the precision-recall (P-R) curve, denoted as AP, provides a measure of the model’s performance.

**Figure 8:**
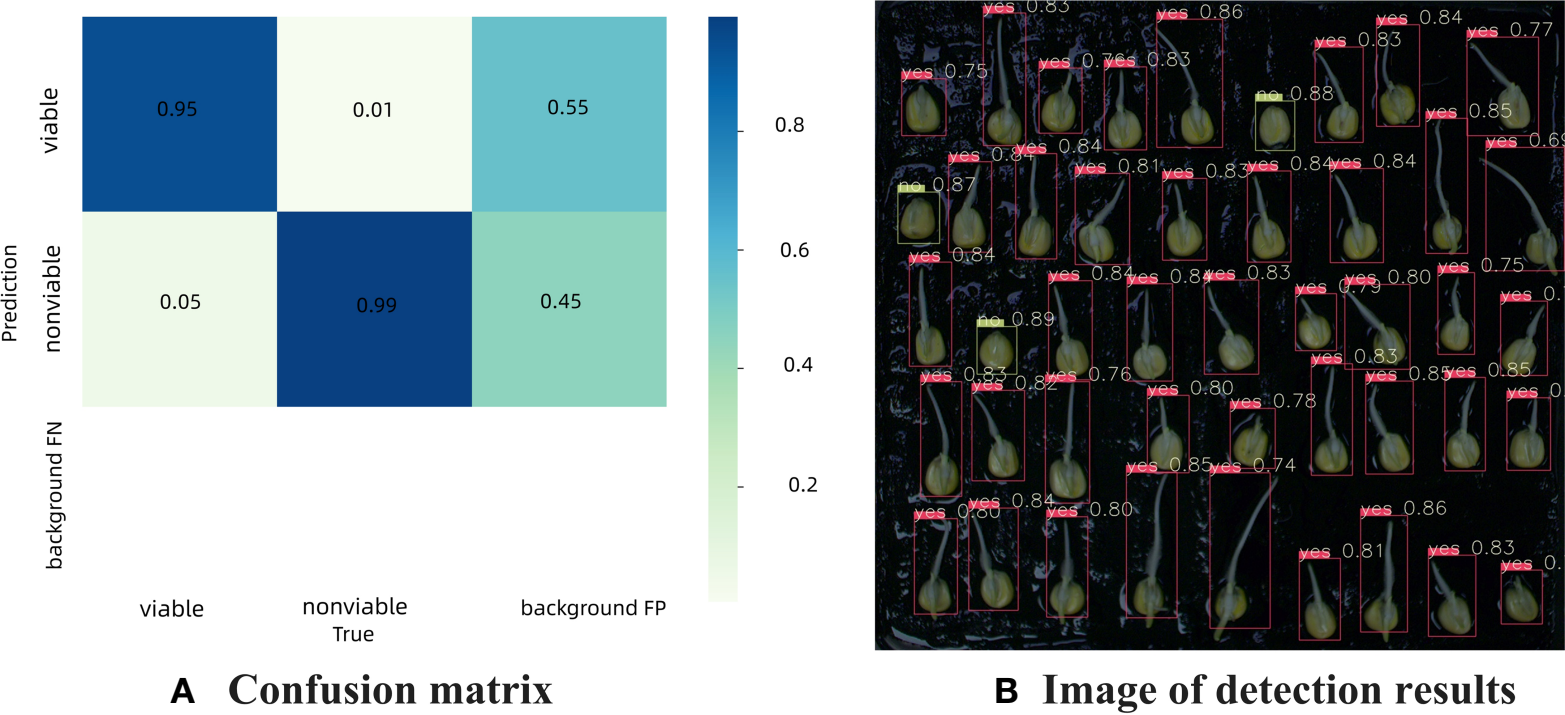
Confusion matrix and detection results of germination maize seed based on YOLOv7 model **(A)** Confusion matrix, **(B)** Image of detection results.

**Figure 8A** is the confusion matrix of germinated maize seed based on YOLOv7 model, which provides a visual representation of the classification performance, showing the counts of true positive, true negative, false positive, and false negative predictions. The detection accuracy was 95% for germinated seeds and 99% for ungerminated seeds, respectively. Background FP refers to the number when the background is erroneously predicted as a target, fortunately there was no background area was incorrectly classified as a target in this study. **Figure 8B** shows the actual detection results of YOLOv7 for discriminating seed germination.

All indicators mean that the model can essentially replace manual observation for determining seed germination status. Therefore, although this method required some time and manpower for data annotation and training, the overall cost was much lower than manual operation, and can provide a reference for rapid detection of seed germination in crops. On the other hand, the algorithm suffered from the problem of duplicate detection in practical applications (Chen et al., 2023a), resulting in some seeds may be simultaneously labeled as germinated and non-germinated. This phenomenon may lead to a misclassification and reduce the practicality and reliability of the algorithm. Hence, future work will focus on improving the algorithm to solve the duplicate detection problem.

### 3.7 Maize seed bud length detection based on Mask R-CNN

The Mask R-CNN model achieved an impressive mAP score of 0.9571, indicating its effectiveness and accuracy in detecting and localizing objects. The mAP is a widely used evaluation metric for object detection models, and a high mAP score indicates that the model performs well in both precision and recall, making it a reliable choice for seed germination analysis. Additionally, the loss value during training decreased significantly, stabilizing around 0.21 from an initial value of 2.61, which is a clear indication of the model’s ability to learn and adapt effectively.

**Figure 9:**
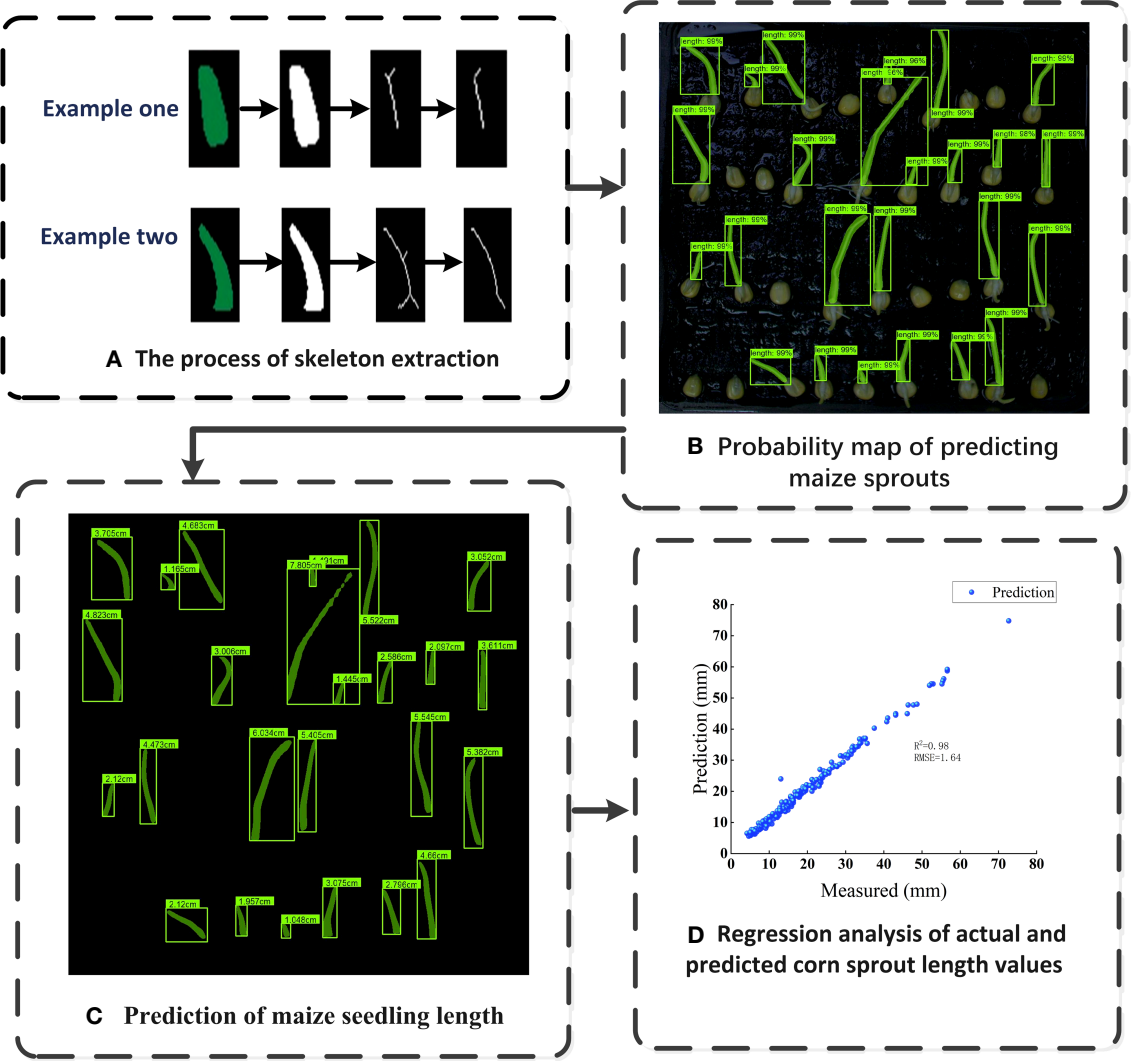
The bud length detection of germinated maize seeds **(A)** The process of skeleton extraction, **(B)** Probability map of predicting maize sprouts, **(C)** Prediction of maize seedling length, **(D)** Regression analysis of actual and predicted corn sprout length values.

**Figure 9A** showcases a successful instance of skeleton extraction for maize seed germination, resulting in a clear main skeleton after removing branches, which allows for accurate calculation of the bud length. The detection results of bud length for germinated maize seeds, depicted in **Figure 9B**, demonstrate Mask R-CNN’s impressive capability to accurately segment the seedlings, even when instances overlap or are occluded. This highlights the superiority of the Mask R-CNN model in instance segmentation tasks, making it a valuable tool for precise analysis of seed germination and growth.

**Figures 9C, D** shows the detection result of bud length with R-squared value of 0.98 and an RMSE of 1.64, demonstrating that the integration of Mask R-CNN model and skeleton extraction method could detect the bud length during seed germination accurately and rapidly. The R-squared value, also known as the coefficient of determination, is a statistical measure that indicates the proportion of the variance in the dependent variable (Bud length in this case) that can be explained by the independent variable (The predicted bud length). Meanwhile, RMSE quantifies the average magnitude of the differences between the predicted bud lengths and the actual observed bud lengths. It is worth mentioning that the bud length of germinated seeds is closely related to their viability (Adebisi et al., 2014). Therefore, the bud length of seeds can be obtained using this algorithm, and the relationship between bud length and viability can be further explored. This not only has important significance for agricultural production but also provides valuable insights for research in other biological fields.


(12) $$\text{SSR }=\;\sum_{i=1}^{n}{\left(Y_{i}-\hat{Y_{i}}\right)}^{2\;}$$

(13) $$\text{SST }=\;\sum_{i=1}^{n}{\left(Y_{i}-\bar{Y}\right)}^{2}\;$$

(14) $$RMSE\;=\sqrt{\frac{1}{n}\sum_{i=1}^{n}{\left(Y_{i}-\hat{Y_{i}}\right)}^{2}}\;\;\;\;\;\;\;\;\;\;\;\;\;\;\;\;\;\;\;\;\;\;\;\;\;\;\;\;\;$$

(15) $$R^{2}=1-\frac{SSR}{SST}$$

In these formulas, SSR (Sum of Squares of Residuals) refers to the regression sum of squares, which represents the sum of squared differences between the predicted values and the true values. On the other hand, SST (Total Sum of Squares) stands for the total sum of squares, representing the sum of squared differences between the true values and their mean.*Y_i_* refers to the actual value of the i-th observation, while $\hat{Y_{i}}$ represents the predicted value of the i-th observation from the regression model. And n denotes the sample size.

## 4 Conclusions

The rapid and successful detection of maize seed viability was achieved by leveraging HSI technology in combination with the multi-scale 3DCNN method. In seed viability detection, the 3DCNN method, which utilizes a limited number of representative spectral bands, was found to learn more complex features and achieve higher accuracy compared to using full-wavelength spectra and machine learning methods. By introducing the multi-scale 3DCNN model, the comprehensive consideration of both spectral and image information enabled a more comprehensive and accurate assessment of maize seed quality. Experimental results demonstrated that the adoption of small block sizes (5 pixels × 5 pixels) significantly improved the accuracy of seed viability detection. Furthermore, the YOLOv7 model and Mask R-CNN model were introduced for germination judgment and bud length detection of maize seeds. Both models exhibited outstanding performance in germination judgment and bud length detection, demonstrating excellent detection capabilities. Based on these exceptional detection results, a novel solution for the rapid detection of maize seed germination and bud length was provided. In brief, this study proposed a reliable and effective method for the evaluation of maize seed viability, providing valuable references for agricultural production and germplasm resource preservation.

## Data availability statement

The raw data supporting the conclusions of this article will be made available by the authors, without undue reservation.

## Author contributions

YF: Conceptualization, Data collection, Data analysis, Writing – original draft, Writing – review & editing. TA, GY, QW, WH, ZW: providing language help. CZ: Resources, Supervision. TX: Resources, Review-editing, Supervision. All authors contributed to the article and approved the submitted version.
